# DiGAlign: Versatile and Interactive Visualization of Sequence Alignment for Comparative Genomics

**DOI:** 10.1264/jsme2.ME23061

**Published:** 2024-03-20

**Authors:** Yosuke Nishimura, Kohei Yamada, Yusuke Okazaki, Hiroyuki Ogata

**Affiliations:** 1 Research Center for Bioscience and Nanoscience (CeBN), Research Institute for Marine Resources Utilization (MRU), Japan Agency for Marine-Earth Science and Technology (JAMSTEC), Yokosuka 237–0061, Japan; 2 Institute for Chemical Research, Kyoto University, Gokasho, Uji, Kyoto, 611–0011, Japan

**Keywords:** synteny map, alignment visualization, comparative genomics, bioinformatics tool, web server

## Abstract

With the explosion of available genomic information, comparative genomics has become a central approach to understanding microbial ecology and evolution. We developed DiGAlign (https://www.genome.jp/digalign/), a web server that provides versatile functionality for comparative genomics with an intuitive interface. It allows the user to perform the highly customizable visualization of a synteny map by simply uploading nucleotide sequences of interest, ranging from a specific region to the whole genome landscape of microorganisms and viruses. DiGAlign will serve a wide range of biological researchers, particularly experimental biologists, with multifaceted features that allow the rapid characterization of genomic sequences of interest and the generation of a publication-ready figure.

As a result of advanced sequencing technologies, the diverse genomic information of cultured and uncultured microorganisms is available through large-scale genomic resources, such as the Genome Taxonomy Database (GTDB) (Parks *et al.*, 2022), proGenomes (Fullam *et al.*, 2023), and microbial and viral genome catalogs derived from metagenomic data (Paez-Espino *et al.*, 2016; Nishimura *et al.*, 2017a; Almeida *et al.*, 2021; Nayfach *et al.*, 2021; Delmont *et al.*, 2022; Nishimura and Yoshizawa, 2022). Comparative genomics has become a fundamental approach to gain insights into microbial ecology and evolution (Brosch *et al.*, 2001; Polz *et al.*, 2013: Kumagai *et al.*, 2018). The identification of conserved sequence regions among microbial genomes facilitates the characterization of evolutionary relationships embedded in genomic structures, such as gene clusters for specific functions, the rearrangement of gene syntenic blocks, and the insertion of mobile genetic elements (Zheng *et al.*, 2011; Cimermancic *et al.*, 2014). With the increasing demand for comparative genomics in the flood of genomic data, several tools for the visualization of a synteny map (*i.e.*, a co-linear alignment of genomic loci) have been developed to characterize microbial genomes (Table 1), such as Easyfig (Sullivan *et al.*, 2011), Artemis Comparison Tool (Carver *et al.*, 2005), Mauve (Darling *et al.*, 2004), genoPlotR (Guy *et al.*, 2010), GenomeMatcher (Ohtsubo *et al.*, 2008), SimpleSynteny (Veltri *et al.*, 2016), AliTV (Ankenbrand *et al.*, 2017), and clinker (Gilchrist and Chooi, 2021). Although these tools provide sufficient functionality and have been widely used in comparative genomics, the following aspects still need improvement: (i) Publication-quality visualization requires extensive preprocessing (*e.g.*, gene prediction, gene annotation, and the orientation arrangement of input sequences), which hinders rapid data visualization. In particular, visualization between circularly permuted or inverted sequences requires extensive manual work to align genomic structures. (ii) When numerous genomic sequences are obtained together, such as in large-scale metagenomic data, time-consuming attempts are required to find an optimized order in which closely related sequences come to neighboring positions. (iii) A synteny map is not suitable for understanding the overall picture of the alignment, particularly when the alignment has complex structures, including inversion, duplication, and translocation. Therefore, it is better to simultaneously use other types of visualization, such as dot plots, which compensate for the weakness of synteny maps. We developed DiGAlign (the **D**ynam**i**c **G**enomic **Align**ment server; https://www.genome.jp/digalign/), a web tool that accepts the nucleotide sequences of microorganisms and viruses as user input and performs gene predictions, gene function predictions, and sequence alignment in‍ ‍both nucleotide and translated amino acid sequences. DiGAlign has unique features to address these issues, such as (i) an automatic sequence position adjustment function including circular permutations and inversions, (ii) a “guide tree” to facilitate the selection of closely related sequences, and (iii) dot plots accompanied by synteny maps. DiGAlign is designed to provide all functionality via a web server so that users do not need to install software or prepare custom scripts to pre-process data. The resulting data are displayed and explored in a web browser, which allows the user to interactively and iteratively refine the visualization in order to produce a publication-quality figure. DiGAlign is provided through GenomeNet (https://www.genome.jp/).

DiGAlign inherits and extends the versatile visualization functions of ViPTree (Nishimura *et al.*, 2017b), a widely used web tool for a viral phylogenomic ana­lysis. Its simple and flexible visualization features have been well received by the virus research community. ViPTree has been widely used to characterize viral phylogenomic relationships and to support proposals for viral taxonomic classification (Low *et al.*, 2019; Turner *et al.*, 2021; Simmonds *et al.*, 2023) through the visualization of phylogenomic trees and geno­mic alignments, which, in turn, have been used in many studies (Okazaki *et al.*, 2019; Yahara *et al.*, 2021). ViPTree takes viral genome sequences as input, performs gene predictions using Prodigal (Hyatt *et al.*, 2010), and computes alignments between input genomes and a prebuilt set of viral reference genomes from the Virus-Host Database (Mihara *et al.*, 2016) using tBLASTx (Altschul *et al.*, 1990). The resulting genomic alignment is visualized interactively in a web browser. The phylogenomic relationship is reconstructed and visualized as a “proteomic tree” based on the similarity score *S*_G_ (Bhunchoth *et al.*, 2016), a similarity metric for a pair of genomes computed as a length-normalized tBLASTx score ranging from 0 (no similarity) to 1 (identical).

DiGAlign is designed to be compatible with both microbial and viral genomes, while ViPTree focuses on viral genomes. The following new features have been implemented in the development of DiGAlign: (i) In addition to a translated amino acid-based alignment using tBLASTx, the user may opt for a nucleotide-based alignment using BLASTn. A nucleotide alignment is suitable for assessing differences between closely related genomes and detecting conserved non-protein coding regions, while a translated alignment is more sensitive for detecting distant homology. (ii) The minimum number of input sequences has been reduced from three to two. (iii) The data size limit has been extended as follows: the maximum number of sequences per submission is 300 and the maximum length of each sequence is 20‍ ‍Mbp. (iv) The user may skip gene and/or function predictions to visualize sequence alignment without gene information with a shorter computation time. (v) The interactive alignment visualization becomes more versatile, with flexibility in the coloring schemes, filtering function of BLASTn/tBLASTx hits, and “mouseover” popup of gene and BLASTn/tBLASTx hit information. The new features of (iii)–(v) are also included in the latest version of ViPTree. An overview of the ana­lysis pipeline is shown in Fig. 1. The visualization functionality of DiGAlign is implemented using D3.js (Bostock *et al.*, 2011), a JavaScript visualization library. The web server is built using Sinatra, a Ruby web framework, and Bootstrap 3, a front-end toolkit that allows for a fluid design that may be browsed even from a mobile phone.

To run DiGAlign, the user only needs to prepare FASTA-formatted nucleotide sequences, which range from specific operon regions to whole genome sequences. On the upload page, the user is asked to select the type of BLAST (BLASTn or tBLASTx), gene predictions (either a prediction using Prodigal, uploading a prepared BED-like formatted gene position table, or no gene information), and gene function predictions (skip or perform GHOSTX (Suzuki *et al.*, 2014) protein similarity searches against GenomeNet nr-aa, a non-redundant protein sequence database merging the sequences of RefSeq, SwissProt, TrEMBL, and GenPept). It is important to note that Prodigal is not designed for eukaryotic sequences. If sequences include those of eukaryotes and eukaryotic viruses, the uploading of pre-computed gene prediction results is recommended. The computation generally takes a few minutes to a few hours, depending on the input data and availability of computing resources. If gene function predictions (*i.e.*, the GHOSTX similarity search), which is a relatively time-consuming process, are skipped, the computation is generally completed within a few minutes. Computing resources are provided by the SuperComputer System, Institute for Chemical Research, Kyoto University.

After the computation is complete, a “session main page” (Fig. 2) is created to review computation details and provide links to the results. The user receives an e-mail notification with a URL to the page. Users may interactively customize different types of visualizations in a web browser, such as the alignment view, the gene table view showing gene annotation results, and the tree view showing the degree of similarity of input sequences through a “guide tree” (Fig. 1). These views are interconnected by hyperlinks embedded in each page, which users may navigate by simply clicking on the links. Visualizations may be explored and fine-tuned with many options implemented as radio buttons, fill-in text fields, and drop-down lists. A typical issue associated with genomic sequence alignment is that input sequences are often inverted and/or circularly permuted unless proper preprocessing has been performed. The alignment view of DiGAlign automatically selects the position and orientation of sequences to clearly visualize complicated alignments, including circular and inverted sequences, a feature that is not commonly available in other tools (Table 1). As implementation details, the automatic adjustment of the genomic sequence alignment is performed depending on the results of BLASTn (or tBLASTx) as follows. The highest scoring high-scoring segment pair (HSP) of the genome pair between the top two genomes is placed in the center, and the third genome is then adjusted so that the highest scoring HSP is vertically aligned between the second and third genomes. The starting position of the third genome (*i.e.*, considering the circular permutation) is selected so that the centers of the second and third sequences are vertically aligned. The remaining genomes are then aligned successively in the same manner as the third genome. DiGAlign also provides download links for visualizations and raw output files of the computation. Visualizations of the tree and alignment views are downloadable from each view page, and raw outputs, including BLASTn/tBLASTx, gene predictions using Prodigal, and protein similarity searches against GenomeNet nr-aa, may be downloaded from the session main page.

DiGAlign generates a guide tree, a similarity score (*S*_G_)-based tree of input sequences, analogous to the proteomic tree in ViPTree. A guide tree provides an intuitive understanding of similarity relationships between many input sequences and is one of the unique features of DiGAlign that distinguishes it from other alignment visualizers. A default order of input sequences in the alignment view (linked from the session main page) follows the order of sequences in the tree; sequences are automatically sorted so that the closest sequences are placed next to each other. The inner nodes of a guide tree provide links to the alignment view of the sequences under the nodes, which is useful for browsing relationships within a closely related subset. If the nucleotide-based alignment mode is selected, the length-normalized BLASTn score is alternatively calculated as *S*_G_, equivalent to the length-normalized tBLASTx score *S*_G_ in the amino acid-based alignment mode. If only two sequences are given as input, a guide tree will not be generated because it is meaningless. A caveat when interpreting a guide tree is that the tree represents the degree of similarity between the input sequences; however, alternative approaches (*e.g.*, a phylogenetic ana­lysis of a specific gene) are more appropriate for inferring accurate evolutionary relationships between the sequences.

Examples of tree and alignment views are shown in Fig. 3 and 4, respectively. Anoxygenic photosynthetic organisms of the class Alphaproteobacteria were used to characterize regions containing photosynthetic gene clusters (PGCs). The sequences of five plasmids and two 100-kb genomic regions encoding PGCs (Brinkmann *et al.*, 2018) were used as input. In both tBLASTx and BLASTn computations, the e-value threshold was fixed at 1e-2. In this example, the similarity search was performed with tBLASTx. The tree view (Fig. 3) displays a guide tree, which shows similarity relationships between the sequences. The appearance of the tree may be modified by changing options in the “tree configurations/downloads” panel. The reconstructed tree (Newick format) and the tree visualization (SVG format) are downloadable from the “download” tab in the panel. When the “show link to alignment” option is selected, as shown in Fig. 3, each of the inner nodes, represented by filled circles in the tree, is linked to an alignment of the sequences in the subtree under the node. The alignment view (Fig. 4), which may be displayed by simply clicking on the filled circle marked with a red arrow in Fig. 3, shows the genomic alignment of four sequences below the subtree. The appearance of the alignment view may be interactively customized by changing the options and clicking a “redraw” button in the alignment configurations panel. In the “Basic parameters” tab, selecting the “auto” button in the “positioning of sequences” option will automatically invert, reposition, and circularly permute the alignment for a clear visualization. In the same tab, thresholds may be set for a selected display of tBLASTx hits in terms of the % identity, hit score, and hit length. The “customize sequences” tab of the configurations panel provides functionality for the detailed tuning of sequence positions, the sequence order, and the deletion and duplication of sequences to customize the sequence set. The alignment in the figure is automatically adjusted by the “auto” button (Fig. 4). As a caveat, the automatic alignment algorithm does not always produce a precise alignment. For example, if two genomes are similar from end to end, the end may be shifted because the algorithm relies only on the highest scoring HSP. In this case, a subsequent manual refinement using the “customize sequences” tab is recommended after the automatic alignment. The dot plot on the left side of the alignment facilitates structural comparisons between sequences because the alignment is not well-suited for understanding the overall structure of the alignment. When gene predictions are performed, each of the predicted genes is indicated by an arrow. When function predictions are performed, a gene label may be displayed for each gene and a link is provided to the page showing the results of the GHOSTX search of genes against the GenomeNet nr-aa database, which contains 433 million non-redundant proteins as of November 2023. If a gene annotation table is provided as an additional input file when sequence data are uploaded, the annotation provided by the table is displayed and the color of each gene arrow is specified. A “mouseover” popup shows information on genes and BLASTn/tBLASTx hits. The user may download the generated alignment visualization as an SVG-formatted file from the “download” tab.

In summary, DiGAlign is a versatile web server tool for visualizing a synteny map of a given set of nucleotide sequences. DiGAlign may be used for a wide range of purposes, from quickly examining the relationship between input sequences to producing a well-organized, publication-quality figure. Due to the challenges associated with dealing with the vast amount of sequence information in the genomic data flood, DiGAlign will markedly increase the efficiency of data exploration to refine the focus of ana­lysis, thereby contributing to the broad field of biological science.

## Citation

Nishimura, Y., Yamada, K., Okazaki, Y., and Ogata, H. (2024) DiGAlign: Versatile and Interactive Visualization of Sequence Alignment for Comparative Genomics. *Microbes Environ ***39**: ME23061.

https://doi.org/10.1264/jsme2.ME23061

## Figures and Tables

**Fig. 1.**
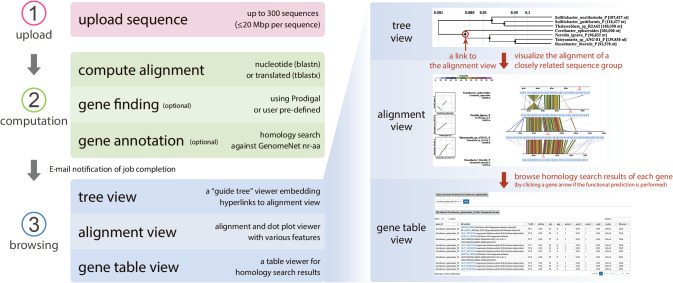
A flowchart of the DiGAlign ana­lysis pipeline. Users upload sequences and select options, and the computation starts immediately after sequence submission if the computation server is available. Upon completion of the computation, users are notified by e-mail with a URL link to the “session main page” where all results may be browsed. The right panel shows the typical workflow for browsing computation results, using hyperlink connections between different types of views.

**Fig. 2.**
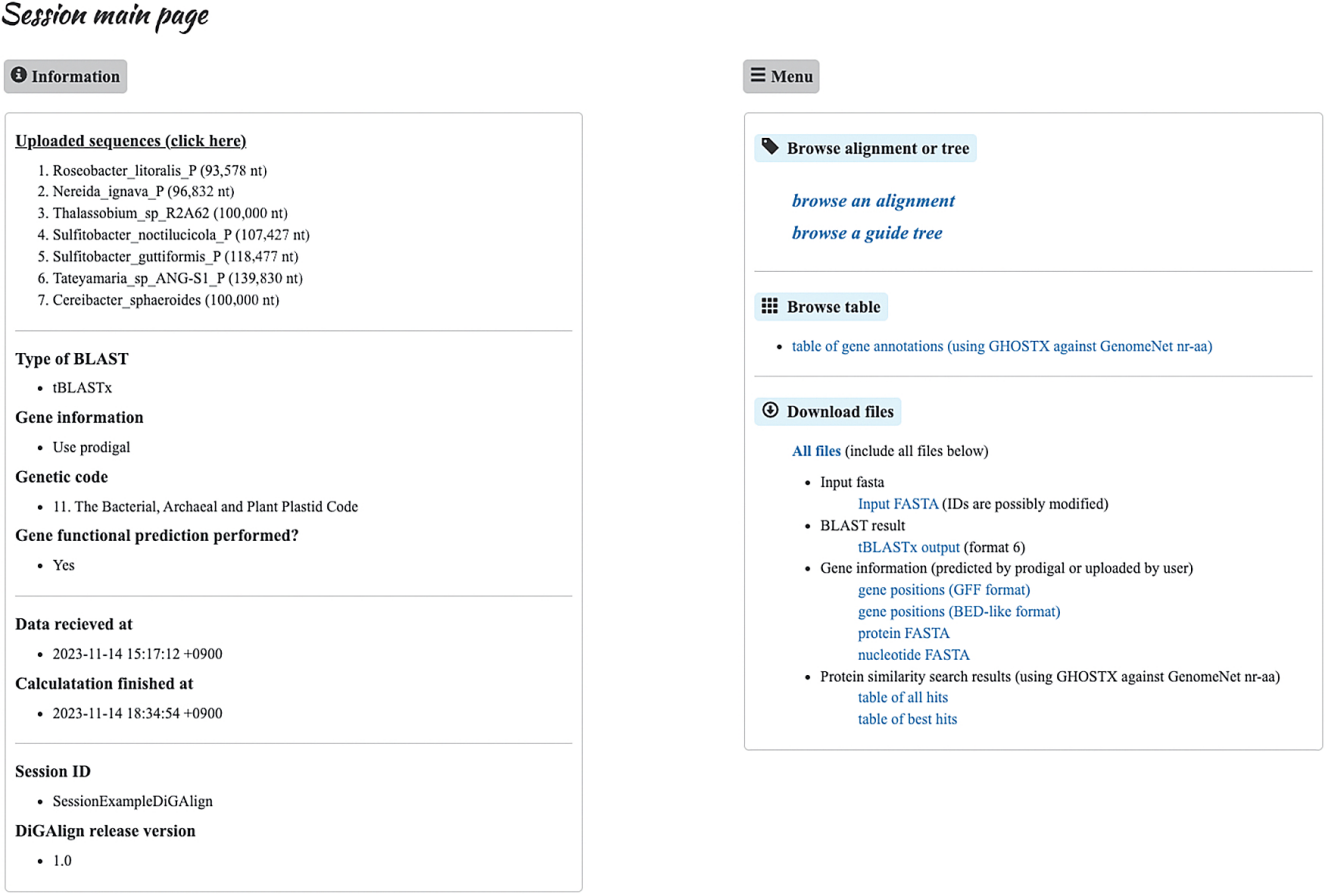
The session main page, created when the computation is complete. The information panel on the left displays the names of the uploaded sequences, the computation options selected on the upload page, and session details, such as computation and expiration dates. The menu panel on the right provides hyperlinks for browsing the results, such as links to the alignment and tree views and gene annotation results. The user may download raw computation results from the panel, such as BLAST hits used for alignment visualization, gene predictions, and gene function predictions.

**Fig. 3.**
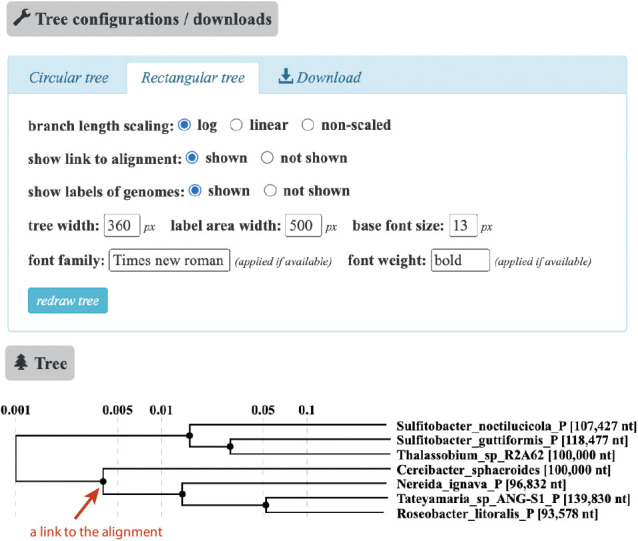
An example of the tree view. The “tree configurations/downloads” panel at the top provides several options for changing the appearance of the tree and the download functionality. The “circular tree” tab provides customization for the circular tree view, while the “rectangular tree” tab provides customization for the rectangular tree view. The “download” tab provides the download link for the tree figure as it appears in the browser in the SVG format. The “tree” panel at the bottom displays a guide tree of the input sequences. Sequence names are displayed to the right of the tree, and plasmid sequence names are shown with the suffix “_P”. If the option “show link to alignment” is set to “shown”, the filled circles in the tree represent hyperlinks to the alignment view, which contains sequences below the subtree. The red arrow highlights a filled circle hyperlink to the alignment view shown in Fig. 4.

**Fig. 4.**
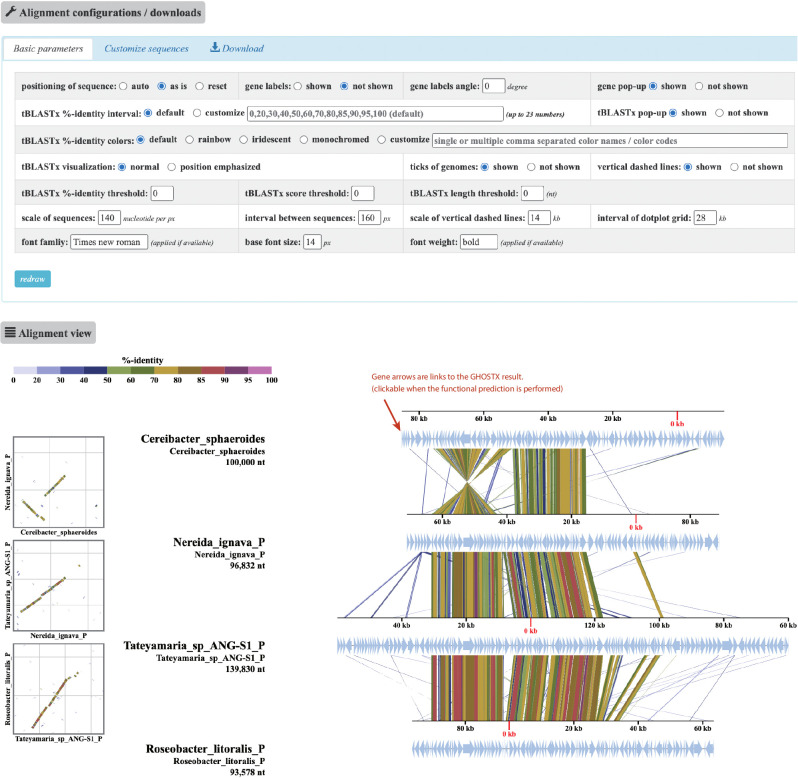
An example of the alignment view. The “alignment configurations/downloads” panel at the top contains three tabs. The “basic parameters” tab provides functions for fine-tuning the visualization, including automatic positioning, alignment color, layout, and scale. The “customize sequences” tab provides functions for manually adjusting genomic positioning and reordering/deleting specific sequences. The “download” tab provides the download link for the alignment figure as it appears in the browser in the SVG format. The “alignment” panel at the bottom provides the visualization of dot plots and a synteny map. Plasmid sequence names are displayed with the suffix “_P”. If function predictions were performed, each gene arrow is a hyperlink to the page showing the result of the GHOSTX search against the GenomeNet nr-aa database.

**Table 1.** Features of DiGAlign and common tools for synteny map visualization.

tool	published year	gene prediction	method for alignment			position adjustment		dotplot visualization	user interface*^1^
			nucleotide	translated		circular permutation	invertion		
Mauve	2004	–	+	–		–	–	–	GUI, CLI
Artemis Comparison Tool	2005	–*^2^	+	+		–	–	–	GUI
GenomeMatcher	2008	–	+	+		–	–	+	GUI
genoplotR	2010	–	+	+		–	–	–	CLI
easyFig	2011	–	+	+		–	+	–	GUI, CLI
SimpleSynteny	2016	–	+	+		+	–	–	Web (server)
AliTV	2017	–	+	–*^3^		–	+	–	Web (local)
clinker	2021	–	–	+		–	–	–	Web (local, server)
DiGAlign	this study	+*^4^	+	+		+	+	+	Web (server)

*1 User interface is categorized as follows. CLI: Command line interface; GUI: Graphical user interface, excluding web interface; Web (local): Web server built on the local computer; Web (server): Web server hosted somewhere. *2 Six-frame translation is available. *3 Alignment data can be provided by the user. *4 Predefined gene information can also be used.

## References

[B1] Almeida, A., Nayfach, S., Boland, M., Strozzi, F., Beracochea, M., Shi, Z. J., et al. (2021) A unified catalog of 204,938 reference genomes from the human gut microbiome. Nat Biotechnol 39: 105–114. 32690973 10.1038/s41587-020-0603-3 PMC7801254

[B2] Altschul, S.F., Gish, W., Miller, W., Myers, E.W., and Lipman, D.J. (1990) Basic local alignment search tool. J Mol Biol 215: 403–410. 2231712 10.1016/S0022-2836(05)80360-2

[B3] Ankenbrand, M.J., Hohlfeld, S., Hackl, T., and Förster, F. (2017) AliTV—interactive visualization of whole genome comparisons. PeerJ Comput Sci 3: e116.

[B4] Bhunchoth, A., Blanc-Mathieu, R., Mihara, T., Nishimura, Y., Askora, A., Phironrit, N., et al. (2016) Two asian jumbo phages, ϕRSL2 and ϕRSF1, infect Ralstonia solanacearum and show common features of ϕKZ-related phages. Virology 494: 56–66. 27081857 10.1016/j.virol.2016.03.028

[B5] Bostock, M., Ogievetsky, V., and Heer, J. (2011) D^3^: Data-driven documents. IEEE Trans Vis Comput Graph 17: 2301–2309. 22034350 10.1109/TVCG.2011.185

[B6] Brinkmann, H., Göker, M., Koblízek, M., Wagner-Döbler, I., and Petersen, J. (2018) Horizontal operon transfer, plasmids, and the evolution of photosynthesis in Rhodobacteraceae. ISME J 12: 1994–2010. 29795276 10.1038/s41396-018-0150-9 PMC6052148

[B7] Brosch, R., Pym, A.S., Gordon, S.V., and Cole, S.T. (2001) The evolution of mycobacterial pathogenicity: clues from comparative genomics. Trends Microbiol 9: 452–458. 11553458 10.1016/s0966-842x(01)02131-x

[B8] Carver, T.J., Rutherford, K.M., Berriman, M., Rajandream, M.-A., Barrell, B.G., and Parkhill, J. (2005) ACT: the Artemis comparison tool. Bioinformatics 21: 3422–3423. 15976072 10.1093/bioinformatics/bti553

[B9] Cimermancic, P., Medema, M.H., Claesen, J., Kurita, K., Wieland Brown, L.C., Mavrommatis, K., et al. (2014) Insights into secondary metabolism from a global ana­lysis of prokaryotic biosynthetic gene clusters. Cell 158: 412–421. 25036635 10.1016/j.cell.2014.06.034 PMC4123684

[B10] Darling, A.C.E., Mau, B., Blattner, F.R., and Perna, N.T. (2004) Mauve: Multiple alignment of conserved genomic sequence with rearrangements. Genome Res 14: 1394–1403. 15231754 10.1101/gr.2289704 PMC442156

[B11] Delmont, T.O., Gaia, M., Hinsinger, D.D., Frémont, P., Vanni, C., Fernandez-Guerra, A., et al. (2022) Functional repertoire convergence of distantly related eukaryotic plankton lineages abundant in the sunlit ocean. Cell Genomics 2: 100123. 36778897 10.1016/j.xgen.2022.100123 PMC9903769

[B12] Fullam, A., Letunic, I., Schmidt, T.S.B., Ducarmon, Q.R., Karcher, N., Khedkar, S., et al. (2023) proGenomes3: approaching one million accurately and consistently annotated high-quality prokaryotic genomes. Nucleic Acids Res 51: D760–D766. 36408900 10.1093/nar/gkac1078 PMC9825469

[B13] Gilchrist, C.L.M., and Chooi, Y.-H. (2021) clinker & clustermap.js: automatic generation of gene cluster comparison figures. Bioinformatics 37: 2473–2475. 33459763 10.1093/bioinformatics/btab007

[B14] Guy, L., Kultima, J.R., and Andersson, S.G.E. (2010) genoPlotR: comparative gene and genome visualization in R. Bioinformatics 26: 2334–2335. 20624783 10.1093/bioinformatics/btq413 PMC2935412

[B15] Hyatt, D., Chen, G.-L., LoCascio, P.F., Land, M.L., Larimer, F.W., and Hauser, L.J. (2010) Prodigal: prokaryotic gene recognition and translation initiation site identification. BMC Bioinf 11: 119. 10.1186/1471-2105-11-119 PMC2848648 20211023

[B16] Kumagai, Y., Yoshizawa, S., Nakajima, Y., Watanabe, M., Fukunaga, T., Ogura, Y., et al. (2018) Solar-panel and parasol strategies shape the proteorhodopsin distribution pattern in marine Flavobacteriia. ISME J 12: 1329–1343. 29410487 10.1038/s41396-018-0058-4 PMC5932025

[B17] Low, S.J., Džunková, M., Chaumeil, P.-A., Parks, D.H., and Hugenholtz, P. (2019) Evaluation of a concatenated protein phylogeny for classification of tailed double-stranded DNA viruses belonging to the order Caudovirales. Nat Microbiol 3: 504–1315. 10.1038/s41564-019-0448-z 31110365

[B18] Mihara, T., Nishimura, Y., Shimizu, Y., Nishiyama, H., Yoshikawa, G., Uehara, H., et al. (2016) Linking virus genomes with host taxonomy. Viruses 8: 66. 26938550 10.3390/v8030066 PMC4810256

[B19] Nayfach, S., Roux, S., Seshadri, R., Udwary, D., Varghese, N., Schulz, F., et al. (2021) A genomic catalog of Earth’s microbiomes. Nat Biotechnol 39: 499–509. 33169036 10.1038/s41587-020-0718-6 PMC8041624

[B20] Nishimura, Y., Watai, H., Honda, T., Mihara, T., Omae, K., Roux, S., et al. (2017a) Environmental viral genomes shed new light on virus-host interactions in the ocean. mSphere 2: e00359-16. 28261669 10.1128/mSphere.00359-16 PMC5332604

[B21] Nishimura, Y., Yoshida, T., Kuronishi, M., Uehara, H., Ogata, H., and Goto, S. (2017) ViPTree: the viral proteomic tree server. Bioinformatics 33: 2379–2380. 28379287 10.1093/bioinformatics/btx157

[B22] Nishimura, Y., and Yoshizawa, S. (2022) The OceanDNA MAG catalog contains over 50,000 prokaryotic genomes originated from various marine environments. Sci Data 9: 305. 35715423 10.1038/s41597-022-01392-5 PMC9205870

[B23] Ohtsubo, Y., Ikeda-Ohtsubo, W., Nagata, Y., and Tsuda, M. (2008) GenomeMatcher: A graphical user interface for DNA sequence comparison. BMC Bioinf 9: 376. 10.1186/1471-2105-9-376 PMC2553346 18793444

[B24] Okazaki, Y., Nishimura, Y., Yoshida, T., Ogata, H., and Nakano, S. (2019) Genome‐resolved viral and cellular metagenomes revealed potential key virus‐host interactions in a deep freshwater lake. Environ Microbiol 21: 4740–4754. 31608575 10.1111/1462-2920.14816

[B25] Paez-Espino, D., Eloe-Fadrosh, E.A., Pavlopoulos, G.A., Thomas, A.D., Huntemann, M., Mikhailova, N., et al. (2016) Uncovering Earth’s virome. Nature 536: 425–430. 27533034 10.1038/nature19094

[B26] Parks, D.H., Chuvochina, M., Rinke, C., Mussig, A.J., Chaumeil, P.-A., and Hugenholtz, P. (2022) GTDB: an ongoing census of bacterial and archaeal diversity through a phylogenetically consistent, rank normalized and complete genome-based taxonomy. Nucleic Acids Res 50: D785–D794. 34520557 10.1093/nar/gkab776 PMC8728215

[B27] Polz, M.F., Alm, E.J., and Hanage, W.P. (2013) Horizontal gene transfer and the evolution of bacterial and archaeal population structure. Trends Genet 29: 170–175. 23332119 10.1016/j.tig.2012.12.006 PMC3760709

[B28] Simmonds, P., Adriaenssens, E.M., Zerbini, F.M., Abrescia, N.G.A., Aiewsakun, P., Alfenas-Zerbini, P., et al. (2023) Four principles to establish a universal virus taxonomy. PLoS Biol 21: e3001922. 36780432 10.1371/journal.pbio.3001922 PMC9925010

[B29] Sullivan, M.J., Petty, N.K., and Beatson, S.A. (2011) Easyfig: a genome comparison visualizer. Bioinformatics 27: 1009–1010. 21278367 10.1093/bioinformatics/btr039 PMC3065679

[B30] Suzuki, S., Kakuta, M., Ishida, T., and Akiyama, Y. (2014) GHOSTX: An improved sequence homology search algorithm using a query suffix array and a database suffix array. PLoS One 9: e103833. 25099887 10.1371/journal.pone.0103833 PMC4123905

[B31] Turner, D., Kropinski, A.M., and Adriaenssens, E.M. (2021) A roadmap for genome-based phage taxonomy. Viruses 13: 506. 33803862 10.3390/v13030506 PMC8003253

[B32] Veltri, D., Wight, M.M., and Crouch, J.A. (2016) SimpleSynteny: a web-based tool for visualization of microsynteny across multiple species. Nucleic Acids Res 44: W41–W45. 27141960 10.1093/nar/gkw330 PMC4987899

[B33] Yahara, K., Suzuki, M., Hirabayashi, A., Suda, W., Hattori, M., Suzuki, Y., and Okazaki, Y. (2021) Long-read metagenomics using PromethION uncovers oral bacteriophages and their interaction with host bacteria. Nat Commun 12: 27. 33397904 10.1038/s41467-020-20199-9 PMC7782811

[B34] Zheng, Q., Zhang, R., Koblížek, M., Boldareva, E.N., Yurkov, V., Yan, S., and Jiao, N. (2011) Diverse arrangement of photosynthetic gene clusters in aerobic anoxygenic phototrophic bacteria. PLoS One 6: e25050. 21949847 10.1371/journal.pone.0025050 PMC3176799

